# A comparison between sphere and rod nanoparticles regarding their *in vivo* biological behavior and pharmacokinetics

**DOI:** 10.1038/s41598-017-03834-2

**Published:** 2017-06-23

**Authors:** Yating Zhao, Yu Wang, Fu Ran, Yu Cui, Chang Liu, Qinfu Zhao, Yikun Gao, Da Wang, Siling Wang

**Affiliations:** 1 Department of Pharmaceutics, School of Pharmacy, Shenyang Pharmaceutical University, 103 Wenhua Road, Shenyang, Liaoning Province 110016 PR China; 2 Chinese PLA No. 463 Hospital, Shenyang, Liaoning Province 110000 PR China; 3 School of Medical Devices, Shenyang Pharmaceutical University, 103 Wenhua Road, Shenyang, Liaoning Province 110016 PR China

## Abstract

In recent years, spherical nanoparticles has been studied extensively on biomedical applications including bioimaging and biosensing, diagnostics and theranostics, but the effect of the shape of nanoparticles has received little attention. In the present study, we designed three different shaped fluorescent mesoporous silica nanoparticles (MSNs), long rod nanoparticles (NLR), short rod nanoparticles (NSR), and spherical nanoparticles (NS) to systematically examine their behavior *in vivo* after oral administration. The results of the *ex vivo* optical imaging study in mice indicated that rod nanoparticles had a longer residence time in the gastrointestinal compared with spherical nanoparticles. The *in vivo* biodistribution showed that all the orally administered MSNs were mainly taken up by the liver, and kidney. NLR had a great capacity to overcoming rapid clearance by the RES and exhibited a longer circulation in the blood than NSR and NS. During renal excretion, the spherical nanoparticles were cleared faster than rod nanoparticles. In addition, it was also found that MSNs can be degraded *in vivo* and NSR were degraded faster than NLR and NS probably owing to their higher specific surface area. The pharmacokinetic results demonstrated that nifedipine(NI)-loaded NLR had a higher bioavailability than NI-loaded NSR and NS.

## Introduction

As the rapid development of science and technology, mesoporous materials have got continuous progresses and it was increasingly applied in some novel areas due to their unique properties^1–4^. Many previous studies have indicated that the size, surface charge, functionalization and shape of nanoparticles can dramatically influence their biological effects *in vivo*. For example, He *et al*. have reported that MSNs with a smaller size and surface PEGylation cannot be easily captured by the reticuloendothelial system (RES) and hav a slower degradation rate^5^. Lee *et al*. have reported that particles smaller than 20 nm are cleared more rapidly than larger 100 nm particles^6^. It has also been reported that the accumulation of particles in target organs and their clearance rate from the body can be influenced by altering the surface charge and size^7–9^. As a novel drug delivery system, the targeted drug delivery of MSNs has also been studied in detail, and MSNs with surface functionalization of different targeting molecules can exhibit increased aggregation in target organs and cells, or escape uptake by the RES^10–13^. In addition, the *in vivo* biological behavior and toxicity of nanoparticles have also been investigated^14–20^. For example, nanoparticles modified by PEG can more easily avoid capture by the liver, spleen, and lungs, resulting in a lower amount of degradation products being excreted in the urine^5, 14^. Also, the toxicity of mesoporous hollow silica nanoparticles (MHSNs) is low when given by intravenous injection^17^.

Recently, aspect ratios (ARs) have attracted more and more attention as these could offer various orientation possibilities in terms of the interaction with cells, overall *in vivo* behavior and tumor penetration^21–29^, resulting in many superior characteristics compared with conventional spherical nanoparticles, such as a higher cellular internalization rate, a longer blood-circulation time, higher catalytic activity, and improved tumor penetration. For example, Huang *et al*. demonstrated that nanoparticles with larger ARs are taken up in greater amounts and were internalized faster by A375 human melanoma (A375) cells^27^. They also found that there were clear particle shape effects involving *in vivo* behavior including biodistribution and clearance, after intravenous injection^15^. However, until now, there have been very few reports on the effects of particle shape on the *in vivo* biological behavior after oral administration. Therefore, the investigation of the behavior *in vivo* of MSNs after oral administration is vitally important and necessary for future potential bioapplications. Recently, Li and co-workers mainly studied the biodistribution, excretion and toxicity of MSNs after oral exposure^30^. However, there are still many unanswered questions in terms of their *in vivo* behaviors. The gastrointestinal retentiveness, the confirmation of degradation products, and pharmacokinetics *in vivo* of drug loaded MSNs are still unclear to date as far as we know. Hence, we systematically examined the *in vivo* characteristics of different shaped MSNs after oral exposure and, in addition, the pharmacokinetic of NI-loaded MSNs were also evaluated. In this paper, we used *ex vivo* optical imaging to qualitatively monitor the retention in the gastrointestinal tract. After the nanoparticles were taken up into the blood from the gastrointestinal tract, the Si content in different organs was measured by inductively coupled plasma-optical emission spectrometry (ICP-OES). Subsequently, the excretion of nanoparticles was investigated by measuring the Si content in feces and urine and the degradation of these MSNs was also studied *in vitro* by simulating physiological conditions to preliminarily explain the mechanism of metabolism. The molybdenum blue method and GFAAS were respectively used to make a qualitative and quantitative detection for Si degradation products. Importantly, the cytotoxicity and gastrointestinal irritation of all three MSNs were also examined. Finally, the shape effect of the three types of MSNs was investigated by HPLC in terms of the pharmacokinetics of the loaded drugs. We believe that the *in vivo* biodistribution of the nanoparticles and the mechanisms of metabolism and excretion determine the suitability of such a novel drug delivery system for use in clinic situations. Hence, we carried out a preliminary and systematic analysis of this and, in addition, we examined why MSNs could improve the oral bioavailability of insoluble drugs and why NLR has a higher oral bioavailability than NSR and NS.

## Materials and Methods

### Materials

Cetyltrimethyl ammonium bromide (CTAB), tetraethyl orthosilicate (TEOS), ammonium hydroxide (NH_4_OH, 28–30%), sodium hydroxide, succinic anhydride, ammonium molybdate, ascorbic acid, citric acid, NaSiO_3_·9H_2_O, sodium dodecyl sulfate (SDS) were obtained from Shan Dong Yu Wang Reagent Company (China). Cy5.5- hydrazide were purchased from Lumiprobe. 4′, 6-diamidino-2-phenylindole (DAPI), 3-(4, 5-dimethylthiazol-2-yl)-2, 5-diphenyltetrazolium bromide (MTT) purchased from Sigma-Aldrich. 2-Amino-2-(hydroxymethyl)-1,3-propanediol (tris), pepsase, trypsase, 3-Aminopropyltriethoxysilane (APTES) were obtained from Aladdin. Raw nifedipine (purity > 99.0%) was obtained from Shenyang Funing Pharmaceutical Company (Shenyang, China). All other chemicals were of analytical grade and used without further purification.

Sprague-Dawley (SD) rats (180–220 g) and Kunming mice (18–22 g) were purchased from the Experimental Animal Center of Shenyang Pharmaceutical University. They were acclimated in the controlled environment (temperature: 22 ± 1 °C; humidity: 60 ± 10% and light: 12 h light/dark cycle) with free access to water and commercial laboratory complete food. Animal experiments were carried out in accordance with the Guide lines for Animal Experimentation of Shenyang Pharmaceutical University and the protocol was approved by the Animal Ethics Committee of this institution.

### Synthesis of the MSNs with different aspect ratios

In this paper, three different shaped MSNs were synthesized by simple and feasible ways. NLR and NSR were synthetized by controlling the addition of NH_3_·H_2_O, they were prepared as follows, briefly, 0.7 g cetyltrimethyl ammonium bromide (CTAB) was dissolved in 200 ml H_2_O, 7 ml and 2.5 ml NH_3_·H_2_O (28–30%) was respectively added with stirring for 30 min to prepare NLR and NSR, 2.4 ml TEOS was then added with vigorous stirring for 5 h at room temperature. The NS (sphere nanoparticles) was synthesized according to the previous paper^31^. 0.5 g CTAB was first dissolved in 240 mL deionized water under vigorous stirring, then 1.8 mL 2 M NaOH (aq) was added. After the mixture was heated to 80 °C for 30 min, 2.5 mL TEOS was added with stirring for 2 h. Finally, three resulting solids were centrifuged and washed with ethanol three times. To remove the template CTAB, the resultant particles were dispersed in 300 ml solution of NH_4_NO_3_ (1 g) and ethanol (100 mL), finally, refluxed for 24 h on 75 °C.

### Preparation of carboxylic acid-functionalized silica nanoparticles

To covalently combine cy5.5-hydrazide with MSNs, the Si-OH firstly should be carboxylated to carry on subsequent amidation reaction. The above resulting silica colloidal was dispersed in 150 ml ethanol and was functionalized by quickly added 0.3 ml of APTES. The mixture were vigorously stirred and refluxed on 70 °C overnight. The obtained MSN-NH_2_ was centrifugation and cleaned by ethanol. Then, it was dispersed in 40 ml DMF, 8 ml of 0.3 M succinic anhydride in DMF was added dropwise. The mixture was stirred for 24 h, the resulting carboxyl functionalized silica nanoparticles (SiO_2_–COOH) were centrifuged and cleaned by ethanol. Finally, the product was dried and sifted.

### Synthesis of fluorescent MSN/COOH-Cy5.5

Cy5.5-hydrazide was reacted with the carboxyl group on the surface of MSN/COOH via EDC/NHS coupling method to fabricate fluorescent nanoparticles for imaging. A total of 30 mg of MSN/COOH was dispersed in 5 mL distilled water and activated by EDAC (20 mg) and sulfo-NHS (20 mg) for 1 h. The nanoparticles were then centrifuged and cleaned to remove excess EDAC/NHS and the water-soluble isourea byproduct. Activated nanoparticles were redispersed in 5 mL distilled water and reacted with 0.06 mg Cy5.5-hydrazide for 12 h. Finally, the nanoparticles were centrifuged and washed with deionized water for three times to remove any unbound Cy5.5-hydrazide. The Cy5.5-labeled nanoparticles were obtained.

### Characterization of the nanoparticles

The morphology and the mesoporous structure of the nanoparticles were performed by Transmission electron microscopy (TEM) (Tecnai G2 F30, FEI, Eindhoven, The Netherlands). N_2_ adsorption–desorption isotherms of bare MSNs were measured by a surface area analyzer (V-Sorb 2800 P, Gold APP Instrument Corporation, Beijing, China). The size distributions and zeta potentials were measured on a Nano-zs90 Nanosizer (Malvern Instruments Ltd., UK) and the samples were dissolved into distilled water before testing. Fourier transform infrared spectroscopy (FT-IR) spectra were recorded on a FT-IR spectrometer (Bruker IFS 55, Faellanden, Switzerland) in the range between 4000 cm^−1^ and 400 cm^−1^. Fluorescence intensity are measurement by Microplate reader (Bio-rad, iMark, America).

### Cellular toxicity

Owing to the MSNs were intragastricly administrated in this paper, Caco-2 cells were selected to investigate the cytotoxicity. The Caco-2 cells were maintained in high glucose Dulbecco’s modified Eagle’s medium (DMEM) containing 10% fetal bovine serum (FBS) and 1% antibiotics (penicillin/streptomycin) and 1% non-essential amino-acid at 37 °C, 5% CO_2_. The MTT assay was applied to investigate the cytotoxicity of the NLR, NSR, NS against Caco-2 cells^32, 33^. In brief, approximately 5 × 10^4^ cells/well were seeded into 96-well plates and incubated for 24 h. The fresh medium containing serial concentrations (50, 100, 250, 500, 1000 μg/mL) of nanoparticles (sterilized by ultraviolet lamp) was added after removing the culture medium. After 24 h incubation, the cells were washed three times with Hanks’ balanced salt solution (HBSS, pH 7.4) to remove particle suspensions or blank culture medium. 20 μL MTT (final concentration 0.5 mg/mL) was added and incubated for another 6 h to quantity the living cells. Then the MTT medium was removed and 200 μL of DMSO was added to dissolve the precipitates. At last, the 96-well plate was placed in a microplate reader (Bio-rad, 680, America) to measure the absorbance at 540 nm and the cytotoxicity was expressed as the percentage of the cell viability compared with the control.

### Gastrointestinal residence time

The *ex vivo* optical imaging were used to investigate the gastrointestinal residence time (GRT) of three different shaped MSNs. Male Kunming mice (18–22 g) were fasted for 12 h before experiments. All three Cy5.5-labeled MSNs were given to mice through oral administration (80 mg/kg), followly, the mice were sacrificed at 40 min, 2 h, 6 h, and 12 hours respectively. The gastrointestinal tract of mice were collected and imaged using an *in*-*vivo* imaging system (FX Pro, Carestream Health, USA).

### Bio-distribution and excretion of different shaped MSNs

It is known that the particle size, shape, surface charge and so on are key factors to affect the biodistribution and excretion *in vivo*. To study the *in vivo* biodistribution of different shaped MSNs after oral administration, male Kunming mice were divided into four groups: control group, NLR-, NSR-, and NS-treated groups. The mice were administered by gavage with different shaped bare MSNs at a dose of 80 mg kg^−1^. Then the organs of mice (including heart, liver, spleen, lung, kidney, intestine) were taken out at 2 h, 24 h, 7 days and were analyzed by ICP-OES. In addition, the feces and urine of mice also were collected and detected.

### *In vitro* biodegradation behavior of MSNs

#### Verification of degradation products

The molybdenum blue method were used to identify the degradation products of MSNs. Relevant studies have shown that silicate ions can react with ammonium molybdate under appropriate conditions converting it into silicomolybdenum yellow heteropoly acid, which can then be converted by a reducing agent into molybdenum blue. Hence, if the degradation products of MSNs are in the form of silicate ions, the above phenomenon would be seen.

#### The quantitative measurement of degradation products in SGF, SIF and SBF

GFAAS were used to detect the amount of degradation products of MSNs in simulated physiological liquid. Simulated gastric fluid (SGF, pH = 1.2), simulated intestinal fluid (SIF, pH = 6.8), an simulated body fluid (SBF, pH = 7.4) were prepared by referring to the Chinese pharmacopoeia. NaSiO_3_·9H_2_O was used to prepare 250 μg/L Si solution. The solution was then automatically diluted by AAS to give a series of concentrations, 25, 50, 75, 100, 125, 150, 175, and 225 μg/L, measurement was carried out, an Si standard curve was obtained. Subsequently, the extracted sample solutions containing degradation products were measured by GFAAS. And the amount of Si degradation products were calculated using the standard curve.

### *In vivo* pharmacokinetics

To study the distinction on the oral absorption of nifedipine (NI)-loaded three types of MSNs, 24 of SD rats (180–220 g) were divided into 4 groups randomly, with 6 in each. Before the test, they were fasted overnight. Commercial NI tablets, NI loaded NLR, NI loaded NSR, NI loaded NS were intragastricly administrated to rats with a dose of 10 mg/kg (that is NI 10 mg/kg). 0.5 ml of blood samples were collected from the eye socket vein at 2, 5, 10, 15, 20, 30, 60, 120, 180, 240, 300 and 360 min after dosing, and then centrifuged at 3000 rpm for 10 min, the plasma samples were extracted and stored at −80 °C. They were analyzed within 15d using HPLC. The pharmacokinetic parameters were calculated using DAS 2.1 version (Boying Corporation, China).

AUC: area under the plasma concentration versus time curve; Cmax: maximum concentration in plasma; D: dose of drug; t_max_: time to C_max_; t_1/2_: half-life.

$${\rm{Rel}}\,{\rm{bioavailability}}={({{\rm{AUC}}}_{0 \mbox{-} \infty })}_{{\rm{a}}}/{({{\rm{AUC}}}_{0 \mbox{-} \infty })}_{{\rm{b}}}\times 100 \% \,({\rm{a}}:\,\mathrm{NI} \mbox{-} \mathrm{loaded}\,\mathrm{MSNs};\,{\rm{b}}:\,{\rm{commercial}}\,{\rm{tablets}}).$$

### Gastric mucosa irritation test

SD rats with body weight of 180–220 g were divided into 4 groups: control group, NLR-, NSR-, and NS-treated groups, they were administrated at a dose of 1000 mg/kg. After 7 days, rats were sacrificed and a 1 cm segment of stomach and duodenum were excised from gastrointestinal tract. Then the samples were washed with physiological saline, fixed with 10% formalin and disposed into 5 μm-thickness sections, then the sections were stained with hematoxylin and eosin (H&E) and observed using a stereomicroscope (Olympus CX 41 Japan).

## Results and Discussion

### Characterization of bare MSNs and carboxylic acid-functionalized MSNs

**Figure 1:**
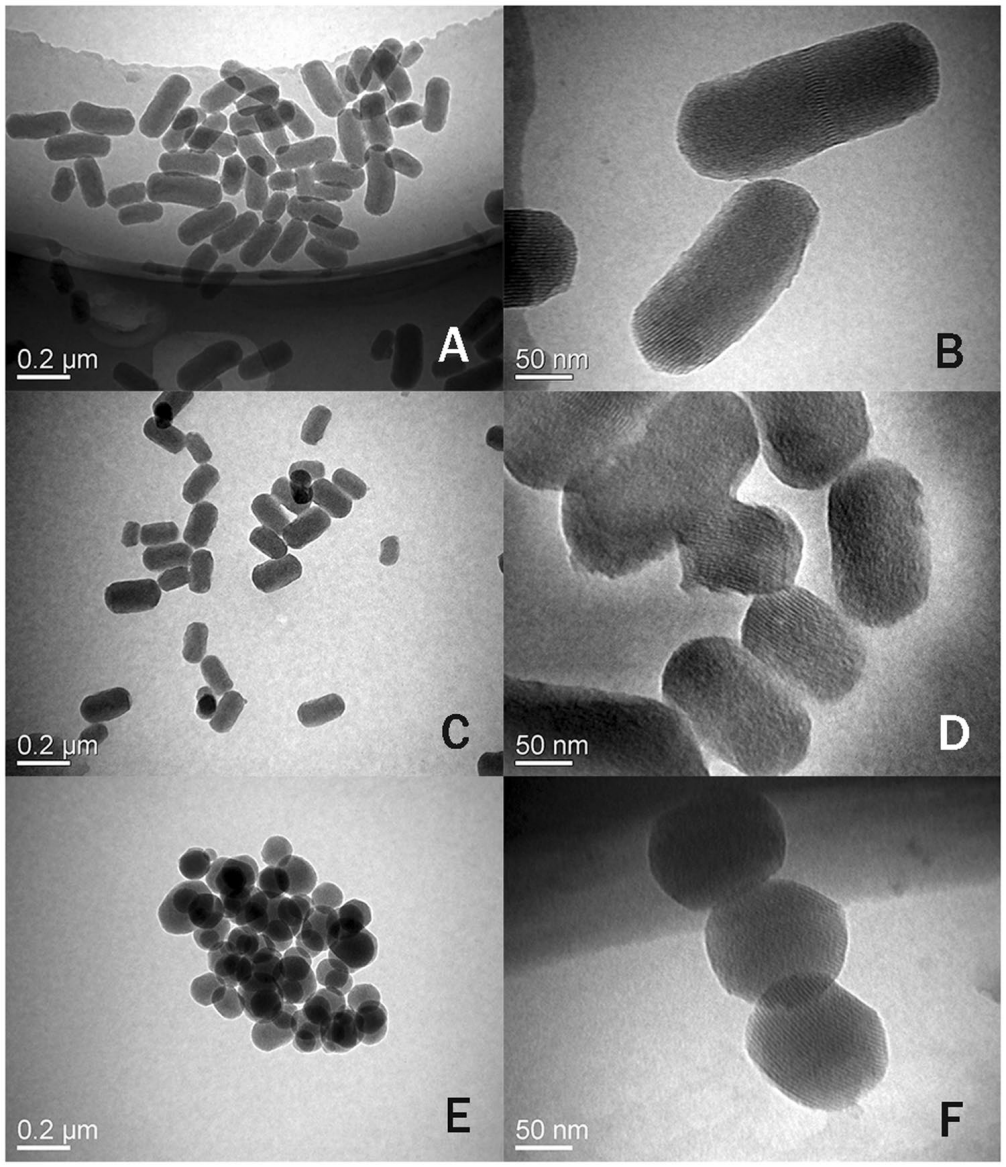
TEM images of NLR (**A**,**B**), NSR (**C**,**D**) and NS (**E**,**F**).

**Figure 2:**
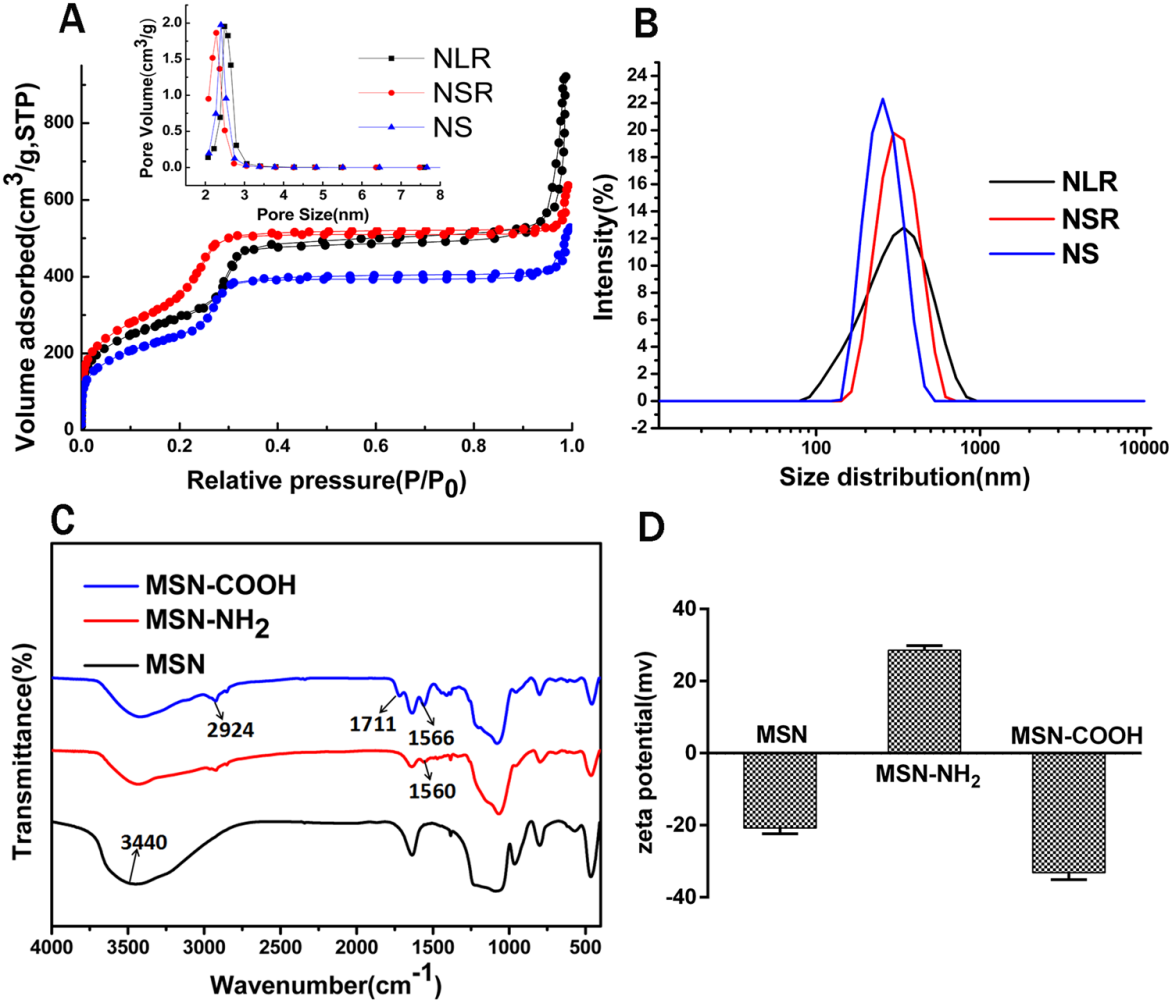
(**A**) Nitrogen adsorption/desorption isotherms and pore size distribution of NLR, NSR and NS. (**B**) Size distribution of NLR, NSR and NS. (**C**) FTIR spectra and (**D**) zeta potentials of MSNs, MSN-NH_2_, MSN-COOH.

To examine the effect of the shape of the nanoparticles on the *in vivo* biological behavior, we prepared three different shaped MSNs but with the same diameter. In addition, they also had roughly the same pore size, chemical composition, surface charge, mosoporous structure. The morphology of the three differently shaped MSNs is shown in the TEM images in Fig. 1. The mean diameter of all types of MSNs was about 150 nm but the AR was different: long rod nanoparticles (NLR) with an AR of ~4 (Fig. 1B), short rod nanoparticles (NSR) with an AR of ~2 (Fig. 1D), and spherical nanoparticles (NS) with an AR of ~1(Fig. 1F). All nanoparticles were monodispersed with high dispersity in aqueous solutions and they all had a highly ordered mesoporous structure. As shown in Fig. 2A and Table 1, the pore size of all three MSNs was approximately similar with an average diameter of about 2.4 nm and the BET surface area was 1092 (NLR), 1506 (NSR) and 933 (NS) m^2^/g, respectively. It is quite clear that the BET surface area of NSR was higher than that of NLR and NS. As shown in Fig. 2B, NS and NSR had a narrower size distribution compared with NLR. In addition, we found the diameter detected by nanosizer was larger than TEM as shown in Table 2, this is because, TEM and nanosizer were two completely different method based on different principle, the former is intuitive method by observing nanoparticles under TEM, while the latter used the principle of dynamic light scattering (DLS) to detect diffusion coefficient of nanoparticles. In addition, due to the formation of the hydration film while nanoparticles were dispersed in dispersion medium, so the size detected by nanosizer was more bigger than TEM.

**Table 1:** The property characterization of NLR, NSR and NS.

Sample	S_BET_ (m^2^/g)	D_p_ (nm)
NLR	1092.3	2.5
NSR	1506.2	2.3
NS	932.7	2.4

**Table 2:** The size of NLR, NSR and NS.

Sample	Diameter (nm)	PDI
NLR	278.1	0.137
NSR	289.0	0.100
NS	250.9	0.039

The formation of MSN-COOH was confirmed by the FT-IR spectrum and zeta potential characterization. The FTIR spectra of unmodified, amino and carboxylic-modified MSN are shown in Fig. 2C. A stretching vibration peak at 3440 cm^−1^ was observed, which was attributed to the hydroxyl group of MSNs (ν_OH_). After modification with APTES, the intensity of the peak at 3440 cm^−1^ was clearly reduced. We believe it was possible that the reaction of APTES and Si-OH masked the peak intensity of Si-OH. Furthermore, a new adsorption band appeared at 1560 cm^−1^ in the spectrum of MSN-NH_2_, which was assigned to the bending vibration of the amino group (δ_NH_) and this was the evidence of the successful modification of MSNs by amino groups. After reaction with succinic anhydride, a distinctive absorption peak at 1711 cm^−1^ was observed. The bands at 1711 cm^−1^ and 2924 cm^−1^ can be respectively assigned to the strong carbonyl stretching vibration (ν_c=o_) and the hydroxyl stretching vibration (ν_O-H_) of the –COOH group. In addition, the band at 1566 cm^−1^ of MSN-COOH can be attributed to the bending vibration of the amide (δ_NH_). This was an indication that succinic anhydride had been successfully grafted onto the surface of MSN-NH_2_. Furthermore, characterization of the zeta potential was also used to confirm the successful modification as demonstrated in Fig. 2D. The potential of bare MSNs with removal of the surfactant CTAB was around −21 mV. After modification with APTES, the zeta potential of the nanoparticles increased to +30 mV. Also, the zeta potential of the nanoparticles fell to −30 mV after reaction with succinic anhydride. In short, the change in zeta potentials also indicated the successful modification of -COOH.

### Cellular toxicity

**Figure 3:**
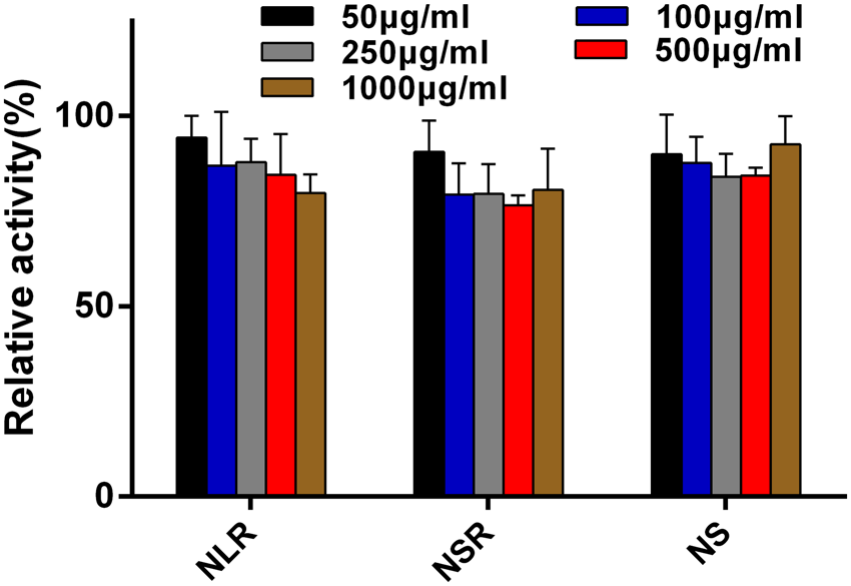
Effect of three types of MSNs on the Caco-2 cell viability at various concentrations from 50 μg/mL to 1000 μg/mL.

Biocompatibility is a major concern when foreign substances, such as nanoparticles, are introduced into cells. So the three different shaped MSNs were incubated with Caco-2 cells for 24 h at various concentrations ranging from 50 μg/ml to 1000 μg/ml and the cell viability of the Caco-2 cells after incubation was evaluated by MTT assay. The results obtained showed that there were no significant changes in cells exposed to nanoparticles compared with control cells that were not exposed to nanoparticles which indicated that the MSNs used in this study were nontoxic or had a very low toxic (Fig. 3). As the dose increased, the cell viability remained nearly constant, even at a concentration of 1000 μg/ml. Such good compatibility can be attributed to a number of factors such as their stable physicochemical properties and good biodegradability. Because the nanoparticles are degraded to silicate ions when they were immersed in physiological liquid (mentioned by follow-up study) and the silicate ions are nontoxic, the nanoparticles were almost completely non-toxic. In addition, it was found that the three types of MSNs exhibited no significant differences in Caco-2 cytotoxicity. After MSNs were confirmed nontoxic to Caco-2 cells, we carried the following animal studies.

### The gastrointestinal residence of differently shaped MSNs

**Figure 4:**
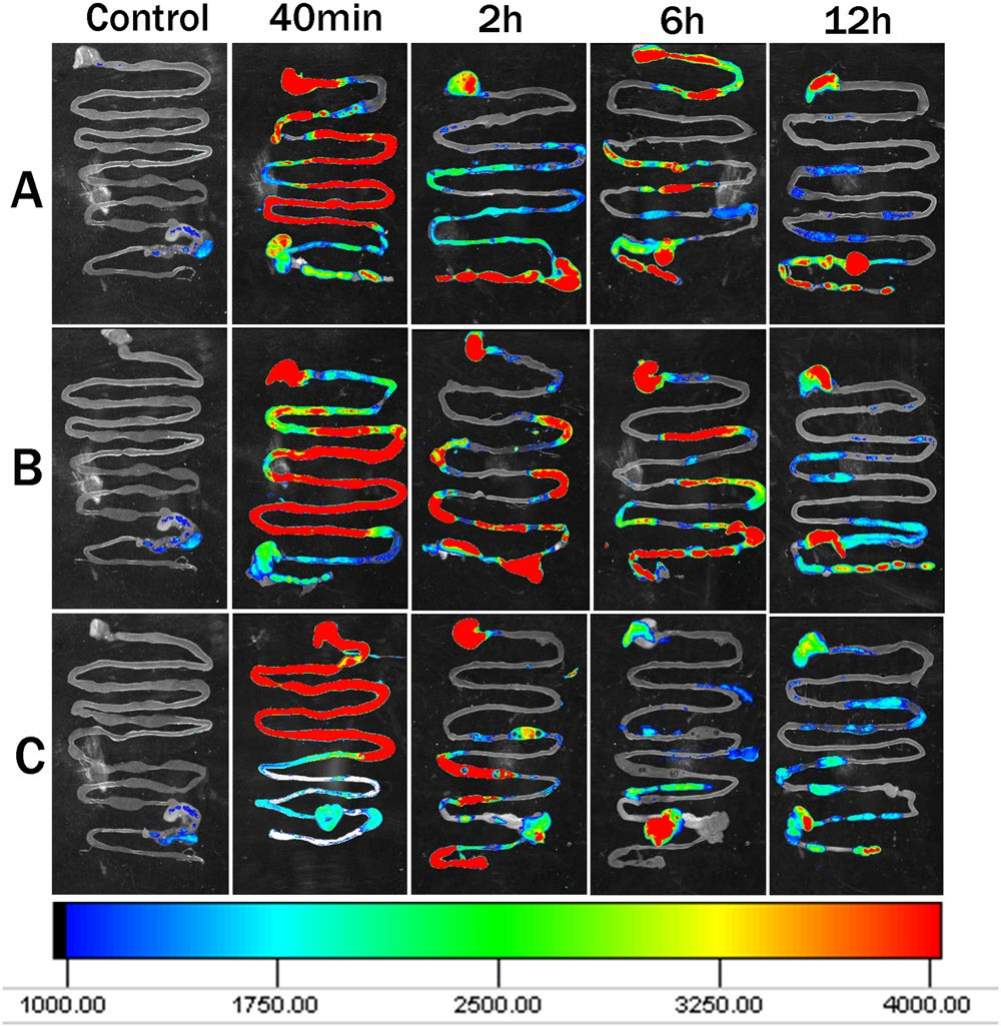
Gastrointestinal of Kunming mice sacrificed at different points after administration of three MSNs (**A**-NLR; **B**-NSR; **C**-NS) were imaged using an *ex vivo* imaging system. The red, green, blue separately indicates the strongest, moderate and weakest fluorescence.

To investigate the shape effect of nanoparticles on gastrointestinal tract retention, we examined the behavior of three types of Cy5.5-labeled MSNs in the gastrointestinal tract using *ex vivo* optical imaging. As demonstrated in Fig. 4, the three types of MSNs all had a long residence time of over 12 h, but the residence time of NS in gastrointestinal was slightly shorter than that of NLR and NSR. At 40 min, the fluorescence signal of all three treated groups was mainly in the stomach, duodenum and jejunum while at 2 h, it was found that fluorescent nanoparticles moved down gradually from the stomach to the intestine. Also, the fluorescence intensity gradually decreased with time. At 6 h and 12 h, there was quite a strong fluorescence in the stomach and intestine for the NLR- and NSR-treated groups, while the fluorescence intensity of the NS-treated groups was weak in comparison. It was found that NS was excreted more rapidly by the feces than NLR and NSR.

### Bio-distribution and excretion of differently shaped MSNs

**Figure 5:**
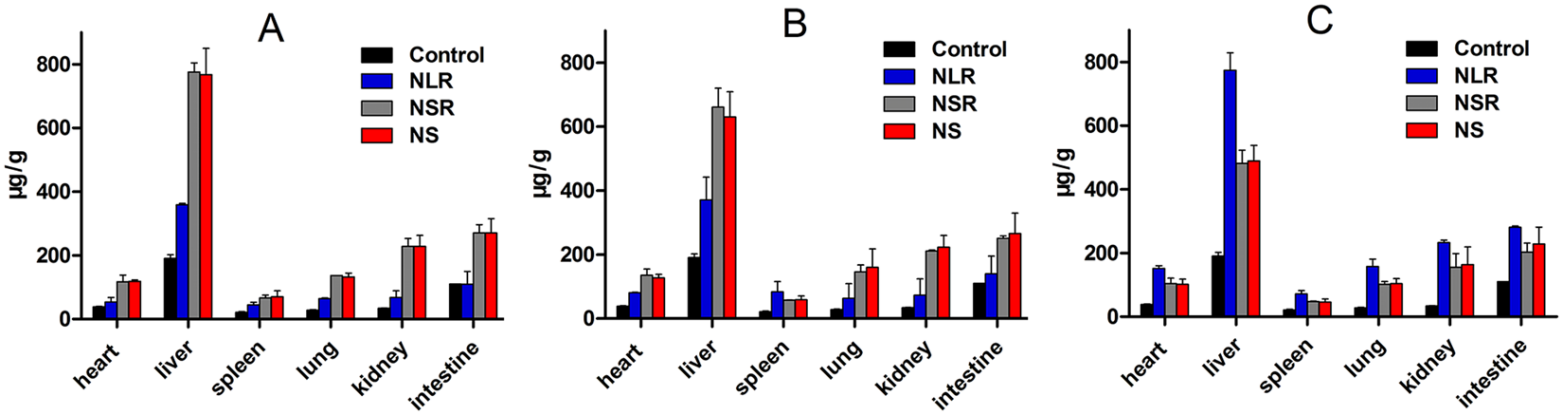
Biodistribution of three different MSNs after oral administration. Si content in heart, liver, spleen, lung, kidney, intestine at (**A**) 2 h, (**B**) 24 h and (**C**) 7 days post-oral.

Studying the biodistribution of differently shaped MSNs is extremely important for the selection of a suitable drug carrier under certain circumstances. To quantify the biodistribution *in vivo* of differently shaped MSNs after oral administration, equivalent doses of all three MSNs were given orally to rats and the Si content in different organs at a fixed time point was analyzed with ICP-OES. Figure 5 shows the biodistribution of nanoparticles in major organs at 2 h, 24 h, and 7d after oral administration, and we found that all three MSNs tended to produce a higher Si content in the liver and kidney after oral exposure compared with other organs. This indicated that the liver and kidney were the major site of nanoparticle accumulation after oral administration. They were also present in the lungs and heart, but taken up by the spleen to a lesser degree. It was also found that the NSR and NS both reached a higher content in all the organs than NLR at 2 h and 24 h, while the NLR attained a higher content in all the organs at 7 days. These results indicated that it was more difficult to remove the NLR from the reticulo-endothelial system (RES) organs and they were protected from macrophages, resulting in a longer blood-circulation. Previous papers have reported that rod- or filament-shaped particles had a longer circulation half-life *in vivo* compared with prototypical spherical particles after intravenous injection^23, 34^. Also, it was confirmed that long-rod micelles exhibited minimal uptake by the RES and long blood circulation half-lives^35^. These findings were consistent with our results and, in addition, we found that the amount of NSR and NS in all organs was reduced with time, demonstrating that the nanoparticles were removed gradually from the body.

**Figure 6:**
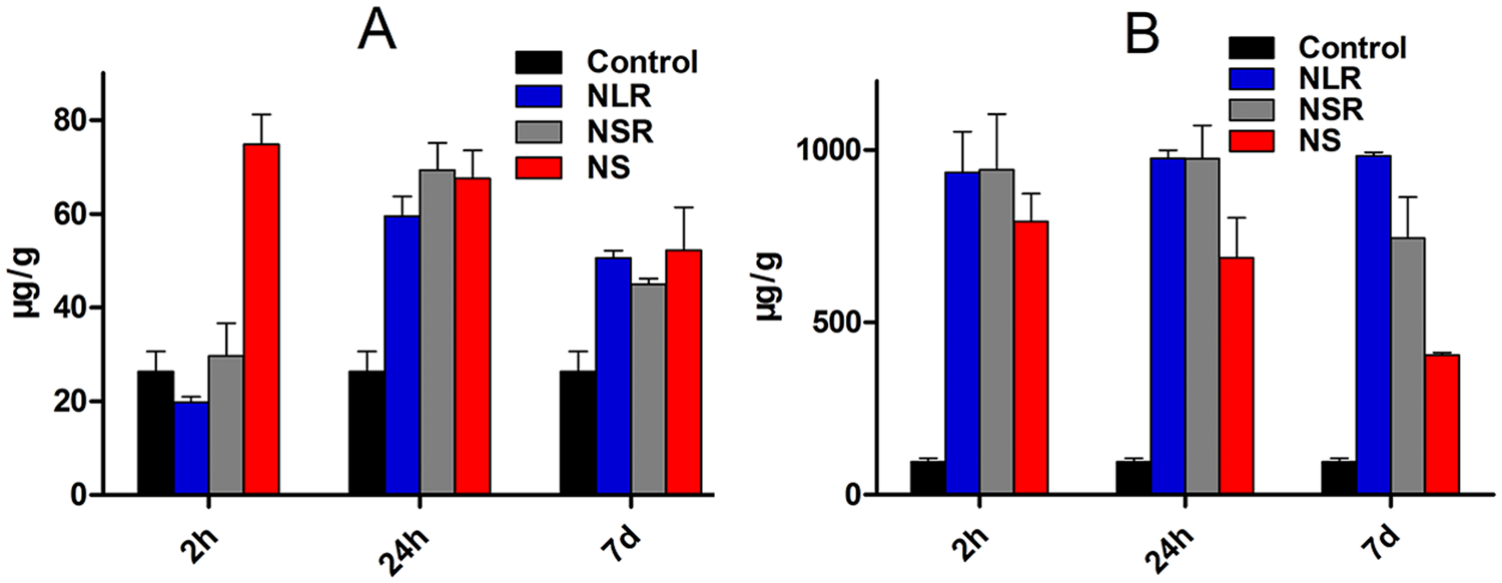
Excretion of three different MSNs after oral administration. Si contents in urine (**A**), feces (**B**) at different time points.

The excretion of the three different shaped MSNs from the body was also analyzed by measuring the Si content in the feces and urine of Kunming mice at different times. As shown in Fig. 6A, MSNs were excreted from urine, which suggested that MSNs are degraded *in vivo* and then cleared by the kidney after oral administration. Also, the three differently shaped MSNs showed different urinary excretion behavior. A number of NS degradation products were excreted from urine at 2 h post-oral dosing and the amount of NS degradation products in urine was reduced at 24 h and 7d. However, the excreted quantities of NLR and NSR degradation products were distinctly less than that of NS at 2 h post-oral dosing while the excreted quantities were emarkedly increased at 24 h and decreased slightly at 7d. These results indicated that rod-shaped MSNs cannot be easily excreted in urine compared with spherical-shaped MSNs. We speculated that the three differently shaped nanoparticles were absorbed into blood from the intestinal tract, and NS were more easily taken up captured by the RES compared with NLR and NSR, then degraded and, finally, excreted in urine. It was also confirmed that rod-shaped nanoparticles had an increased blood-circulation time than spherical nanoparticles. In addition, it was also found that a large amount of Si was present in feces at 2 h and 24 h as shown in Fig. 6B, indicating that the most of the MSNs were mainly excreted from the body in the feces instead of the urine, regardless of the particle shape. Generally speaking, it is normal that the amount of Si in feces at 7 days would be reduced drastically, while the Si content was not significantly reduced. We believe that the nanoparticles and their degradation products underwent the process of enterohepatic circulation. In detail, some intact MSNs or their degradation products were absorbed through the intestinal mucosa and entered into the systematic circulation and, subsequently, the nanoparticles were degraded in body fluids. Both undegraded Si nanoparticles and their degradation products were all mainly excreted from the body via the liver. After being captured by the liver, they were excreted into the intestines by the bile. At that moment, some of the Si nanoparticles were reabsorbed by intestinal epithelial cells and other nanoparticles were excreted from the body via the feces. Hence, the Si content of all three MSNs at 7d was not significantly reduced.

### *In vitro* biodegradation of the MSNs

An in depth understanding of the degradability of MSNs is significant for the safe use of an ideal drug carrier in various biomedical applications. Previous papers have reported that the Si nanoparticles could be degraded in simulated physiological liquid^36–39^. However, to the best of our knowledge, the degradation behavior of MSNs in animals after oral exposure has rarely been studied and a method to verify the degradation products of silica nanoparticles *in vivo* has not been found. Hence, the aim of this study was to identify the degradation products of silica nanoparticles and establish a new feasible method for the quantitative determination of MSNs degradation products in SGF, SIF and SBF after oral administration. In this paper, the molybdenum blue method and GFAAS were respectively used to make a qualitative and quantitative detection for Si degradation products and these two methods have never before been reported in previous literature.

**Figure 7:**
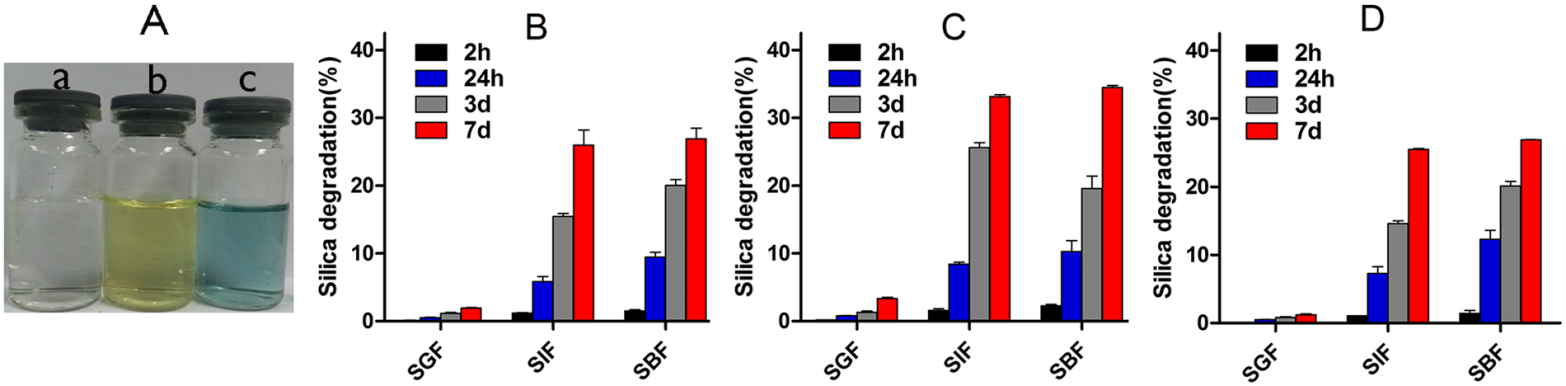
(**A**) The verification of degradation products. And Quantitative analysis of the degradation products of NLR (**B**), NSR (**C**), NS (**D**) in SGF, SIF and SBF at different points by GFAAS.

As the degradation of MSNs in SGF an example, Fig. 7A(a) shows clear solutions of the degradation products. Figure 7A(b) is the reaction product of the degradation products and ammonium molybdate. Figure 7A(c) is the final product after the addition of reductant. As shown in Fig. 7A, the degradation products of MSNs reacted with ammonium molybdate under appropriate conditions take the form of silico-molybdenum yellow as shown in Fig. 7A(b) and can be converted by reducing agent into silico-molybdenum blue as shown in Fig. 7A(c). From above phenomenon, it was confirmed the degradation products of MSNs were silicate ions.

The degradation behavior was found to be related to the inherent physicochemical properties of MSNs, as well as the pH, iron and serum^36^. We have confirmed the degradation products were silicate ions, followlly, the amount of degradation products of MSNs would be detected by GFAAS. As shown in Fig. 7B–D, three MSNs exhibited the slowest degradation in SGF and the fastest degradation in SIF and SBF regardless of the particle shapes, we speculated it was mainly caused by various pH. In detail, all three types of MSNs began to degrade during the first 2 h in SIF and SBF, while there was no obvious degradation in SGF. As time went on, the amount of degradation products steadily increased. At 24 h, the degradation ratio of NLR, NSR and NS was 0.5%, 0.8% and 0.5% in SGF, 5.8%, 8.4%, 7.2% in SIF and 9.4%, 10.3%, 12.3% in SBF. At 7d, the degradation ratio of NLR, NSR and NS was 1.9%, 3.3% and 1.2% in SGF, 26.0%, 33.1%, 25.5% in SIF and 26.9%, 34.5%, 26.9% in SBF. We found that NSR was degraded faster than NS and NLR, and this was probably due to their relative larger specific surface area for accessing bioactive components in the physiological medium. Due to degradation reaction is an interfacial reaction, the lager the surface area of carriers contacted with physiological liquid, the faster the degradation rate. The gastrointestinal tract (GIT) is the main pathway for the absorption of carriers for oral administration and the MSNs could be absorbed into blood by the GIT and, so, investigating the degradation behavior of MSNs in SGF, SIF and SBF is extremely important. From the above results, we speculated that the MSNs began to be degraded when they moved to the gastrointestinal tract and they continued to be degraded when they were taken up into the blood by the intestine after oral administration.

### The pharmacokinetic study of drug-loaded MSNs

**Figure 8:**
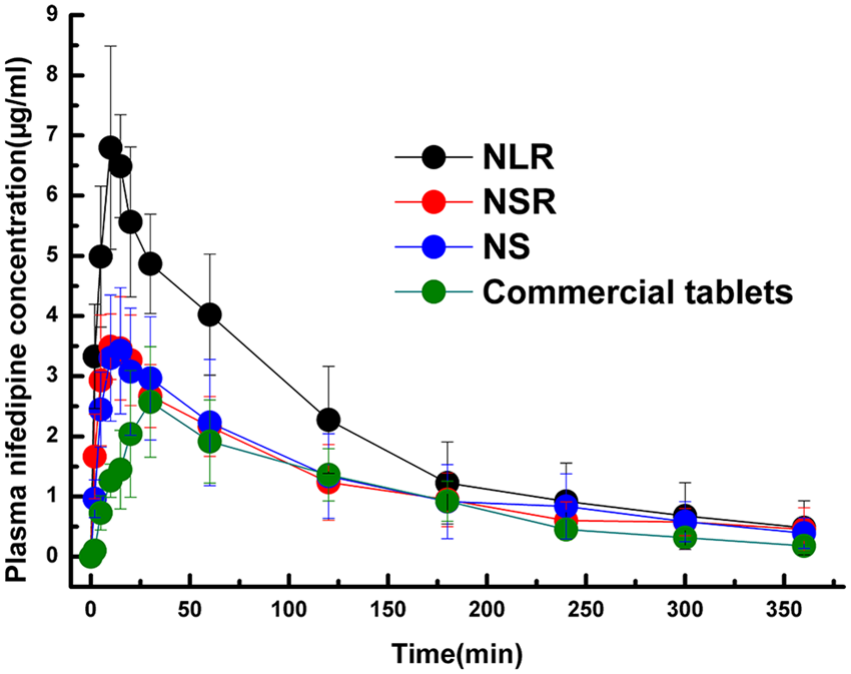
Plasma concentration-time profiles of NI after gavage of the drug loaded three different shaped MSNs and commercial NI tablets in rats. Results were expressed as mean ± SD (n = 6).

It is extremely important to investigate the shape effect of mesoporous silica nanoparticles on pharmacokinetics after oral administration. The plasma drug concentration-time curve of NI after oral administration and the relevant pharmacokinetic parameters are given in Fig. 8 and Table 3, respectively. It was demonstrated that the AUC_0-∞_ of NI-loaded NLR was more than 2.1-fold that of commercial NI tablets, the AUC_0-∞_ of NI- loaded NSR was more than 1.6-fold of that of commercial NI tablets, and the AUC_0-∞_ of NI-loaded NS was more than 1.4-fold that of commercial NI tablets. These results indicated that three shaped MSNs could all improve the bioavailability of NI compared with that of commercial tablets. In addition, the Tmax of the three NI loaded MSNs was shorter than that of commercial tablets, and it only took about 10 min for MSNs to reach Cmax, while commercial tablets took about 30 min. In conclusion, NI-loaded three differently shaped MSNs resulted in a shorter Tmax and higher AUC. These results may be due to the following reasons. Firstly, the drug was present in mesoporous silica pore canals in the non-crystalline state, which was attributed to the mesoporous structure. Secondly, these three types of MSNs all have an increased residence time in the GI of over 12 h, enough time make the drug achieve a sufficient release. Thirdly, the NI-loaded MSNs could be taken up by the intestine into blood, which is another reason for the improved oral bioavailability. On the one hand, the MSNs loading drug system remained outside the intestinal mucosa and gradually released the drug while, on the other hand, the MSNs carrying drug were absorbed by intestinal epithelial cells, and the drug was gradually released in the blood. Fourthly, intuitively, the increased drug bioavailability was also related to the degradation behavior of MSNs. When the carriers were immersed in SGF, SIF and SBF, they began to be degraded and the structure of the MSNs collapsed, allowing the loaded drugs to be completely released. This was another factor contributing to the increased bioavailability. Regarding the shape effect, there was an obvious difference in bioavailability for these three differently shaped MSNs. The relative bioavailability of the NLR loading drug system was highest (212.8%), followed by the NSR loading drug system (157.1%) and the NS loading drug system was the lowest (141.8%). The relative bioavailability of the NLR loading drug system was much higher than that of NSR and NS. However, there was no marked difference in the relative bioavailability of the NSR and NS loading drug system. This result may be due to the following reasons. On the one hand, due to its longer residence time than NS, the carriers possessed enough time to stay outside the intestinal mucosa and gradually released the drugs. On the other hand, NLR possessed longer blood-circulation lifetime after absorbed by intestine than NSR and NS, it was removed as little as possible by RES before the drug fully released in the blood. This two factors interacted together and make it have a highest relative bioavailability”.

**Table 3:** Pharmacokinetic parameters of commercial tablets, NI loaded NLR, NI loaded NSR, NI loaded NS after intragastric administration in rats, mean ± S.D., n = 6.

Parameter	Commercial tablets (10 mg/kg p.o.)	NLR (10 mg/kg p.o.)	NSR (10 mg/kg p.o.)	NS (10 mg/kg p.o.)
AUC_(0-∞)_ (mg·h/L)	383.9 ± 84.1	817.1 ± 231.8	603.3 ± 205.6	543.2 ± 211.8
C_max_ (mg/L)	2.7 ± 1.0	7.0 ± 1.4	3.8 ± 0.8	3.5 ± 1.1
T_max_ (min)	28.3 ± 4.1	12.5 ± 2.7	12.5 ± 5.2	17.5 ± 6.9
t_1/2_ (min)	84.7 ± 39.6	93.2 ± 43.9	197.1 ± 119.0	150.0 ± 82.0
Rel. bioavailability	100.0%	212.8%	157.1%	141.8%

### Gastric mucosa irritation test

**Figure 9:**
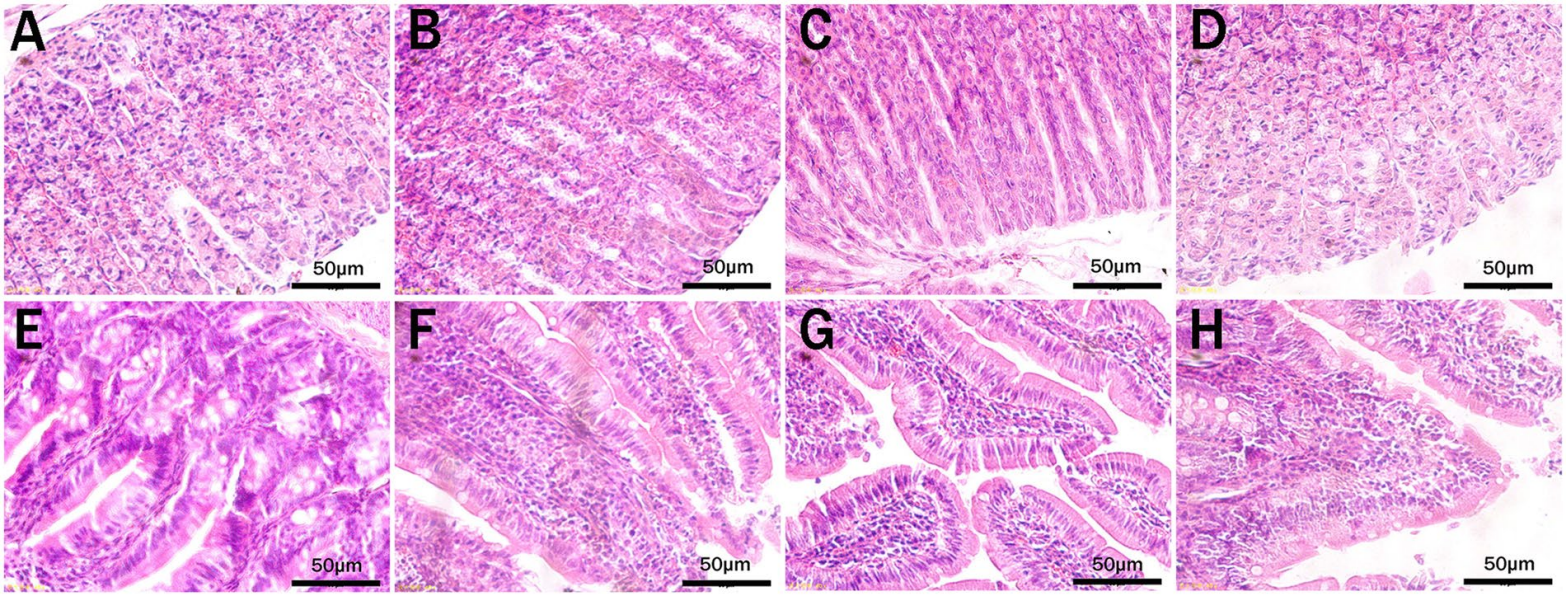
Morphology of rats’ gastric mucosa (**A**–**D**) and duodenum mucosa (**E**–**H**) after oral administration of physiological saline (**A**,**E**), NLR (**B**,**F**), NSR (**C**,**G**), NS (**D**,**H**).

Gastrointestinal irritation evaluation is extremely important when nanoparticles are given orally. Accordingly, to ensure that carriers are safe and effective, a histological evaluation of the gastrointestinal tract was carried out. Figure 9 shows microscope images of slices of stomach and duodenum from SD rats given normal saline and differently shaped MSNs after 7 days. If nanoparticles produce significant tissue toxicity, the following phenomena would be observed, such as stomach mucosal bleeding and atrophy, and intestinal epithelial inflammatory cell infiltration, hyperemia and edema. While compared with the blank control, the treated groups showed no acute pathological changes and no histopathological lesions or hyperemia were observed during the microscopic examination, which indicated that three differently shaped MSNs did not cause any significant gastrointestinal toxicity and inflammation *in vivo*; Therefore, it was concluded that all three differently shaped MSNs exhibited good tissue compatibility and could be used for oral administration.

## Conclusion

Our results showed that the particle shape is a significant factor that needs to be explored for the design of an effective nanocarrier for medical use. It was demonstrated that the *in vivo* behavior and pharmacokinetics of MSN were greatly influenced by the shape. The NLR not only achieved a longer blood-circulation, but also had a highest bioavailability compared with NSR and NS. The mechanism for the increased bioavailability was fully discussed with reference to previous experimental results. NLR has a great potential to improve the oral absorption of drugs with poor bioavailability, and our results provide some useful information for choosing a suitable carrier for use in clinical situations. For example, we can take advantage of its long blood-circulation to design drug delivery systems for an increased drug effect over time. Also, we established two new ways to make a qualitative and quantitative detection for Si degradation products.
